# A Highly Integrated and Diminutive Fluorescence Detector for Point-of-Care Testing: Dual Negative Feedback Light-Emitting Diode (LED) Drive and Photoelectric Processing Circuits Design and Implementation

**DOI:** 10.3390/bios12090764

**Published:** 2022-09-16

**Authors:** Yue Wang, Yile Fang, Haoran Liu, Xiangyi Su, Zhu Chen, Song Li, Nongyue He

**Affiliations:** 1 State Key Laboratory of Bioelectronics, School of Biological Science and Medical Engineering, Southeast University, Nanjing 210096, China; 2 Economical Forest Cultivation and Utilization of 2011 Collaborative Innovation Center in Hunan Province, Hunan Key Laboratory of Green Chemistry and Application of Biological Nanotechnology, Hunan University of Technology, Zhuzhou 412007, China

**Keywords:** fluorescence detection, LED drive circuit, photoelectric processing circuit, point-of-care testing, real-time fluorescence PCR

## Abstract

As an important detection tool in biochemistry, fluorescence detection has wide applications. Quantitative detection can be achieved by detecting fluorescence signals excited by excitation light at a specific wavelength range. Therefore, the key to fluorescence detection is the stable control of the excitation light and the accurate acquisition of weak photoelectric signals. Moreover, to improve portability and instantaneity, devices are developing in miniaturization and integration. As the core of such devices, fluorescence detectors should also have these features. Under this circumstance, we designed a highly integrated and diminutive fluorescence detector and focused on its excitation light driving and photoelectric signal processing. A current–light dual negative feedback light-emitting diode (LED) driving circuit was proposed to obtain constant current and luminance. In addition, a silicon photodiode (PD) was used to receive and convert the fluorescence signal to an electric signal. Then, amplifying, filtering, and analog-to-digital (A/D) converting were applied to make the detection of weak fluorescence signals possible. The test results showed that the designed circuit has wonderful performance, and the detector shows good linearity (R^2^ = 0.9967) and sensitivity (LOD = 0.077 nM) in the detection of *fluorescein sodium* solution. Finally, a real-time fluorescence polymerase chain reaction (real-time PCR) of *Legionella pneumophila* was carried out on a homemade platform equipped with this detector, indicating that the detector met the requirements of real-time PCR detection.

## 1. Introduction

The accurate measurement and quantification of biological and chemical substances have very important roles in applications such as environmental monitoring, clinical diagnosis, and pathogen detection [1,2,3,4,5,6]. Commonly used biochemical detection methods include optical detection, mass spectrometry detection, electrochemical detection methods, and electrochemiluminescence detection. Optical detection measures the absorbance or luminescence properties of the sample; compared with mass spectrometry detection and electrochemical detection, it is easy to operate, free from electromagnetic interference, and stable under severe pressure and temperature [7,8,9,10,11,12,13,14]. Optical detection mainly includes chemiluminescence, absorption light detection, and fluorescence detection. Chemiluminescence detects the content of substances by measuring the intensity of the light signal generated by the sample’s own reaction. It does not require excitation by a light source but has a slower response because the detection signal comes from chemical reactions [15,16,17,18,19,20,21]. Absorption light detection is a method based on the selection of a specific spectrum for the substance to be measured, which is simple in structure and easy to operate but has the disadvantage of low sensitivity [22]. Fluorescence detection can quantitatively and qualitatively analyze targets by exciting samples to emit fluorescence with high sensitivity and accuracy [23,24]. Compared with chromaticity or absorbance-based detection techniques, fluorescence signals are measured directly without comparison to a reference beam, making it more attractive in field applications with small quantities of biochemical samples [25,26].

Because of their high sensitivity and selectivity, fluorescence-based portable detection instruments are widely used in various fields, such as spectrometers, electronic tongues for water quality monitoring, and real-time PCR instruments [27,28]. Wilkes et al. [29] developed a spectrometer with 1 nm resolution using conventional optics combined with a Czerny–Turner monochromator configuration. However, it requires relatively expensive optics and is difficult to achieve a compact form factor. Shin et al. [30]. reported a handheld phytoplankton detector using different excitation lights to selectively excite different types of phytoplankton, which can detect and distinguish different mixtures of green and cyanobacterial species. Currently, a portable fluorescence detector is a suitable option for transferring fluorescence-based diagnostics to field applications. For example, Kozma et al. [31] designed a handheld fluorescence microarray reader using a 635 nm laser diode as the excitation source and selectively measured fluorescence by placing optical elements on the CCD image sensor. Katzmeier et al. [32]. reported a pocket-sized fluorescence detector for point-of-care testing (POCT). An LED with a peak wavelength of 466 nm was used as the excitation light source, and a cadmium sulfide light-dependent resistor was selected as the photodetector to measure the fluorescence emission of the paper strip. Although the technology of fluorescence instrumentation is very mature and advanced, there are still challenges in applying it to a portable fluorescence detection system. The challenge is to achieve sufficiently high sensitivity, small size, and low power consumption while foregoing expensive optical components. Herein, we present an integrated and diminutive fluorescence detector with high sensitivity that can be applied to POCT. This detector has a high-power LED for exciting and a photodiode (PD) for acquiring fluorescence signals. For the detector, a stable excitation source and an accurate photoelectric signal are the focuses and difficulties of the precise measurement of weak fluorescence signals [33]. Therefore, this study focused on high-power LED driving and photoelectric signal processing; a current–light dual negative feedback driving circuit was designed to achieve a stable excitation light, and the signal–noise ratio was improved by amplifying and filtering the fluorescence signal at the detection side.

The dual negative feedback circuit in this paper showed superior stability. The average LED working voltage value was 1.433 V, with a peak-to-peak value of 0.021 V and a fluctuation of ±0.7%. The fluctuation within 5 s was 0.7 μW ± 0.08%, and the fluctuation within 1 h was less than 2 μW. In addition, a *fluorescein sodium* concentration gradient test and real-time fluorescence PCR test for *Legionella pneumophila* were performed. The detector had a high sensitivity with the limit of detection (LOD) of 0.077 nM for *fluorescein sodium,* and it exhibited a linear response in a concentration ranging from 0.05 to 400 nM and a correlation coefficient of R^2^ = 0.9967. As for the real-time PCR test, the results indicated that the detector met the demands of the real-time PCR assay. The portability and low cost of our detector render it useful in point-of-care applications.

## 2. Materials and Methods

### 2.1. Structure of the Fluorescence Detector

**Figure 1:**
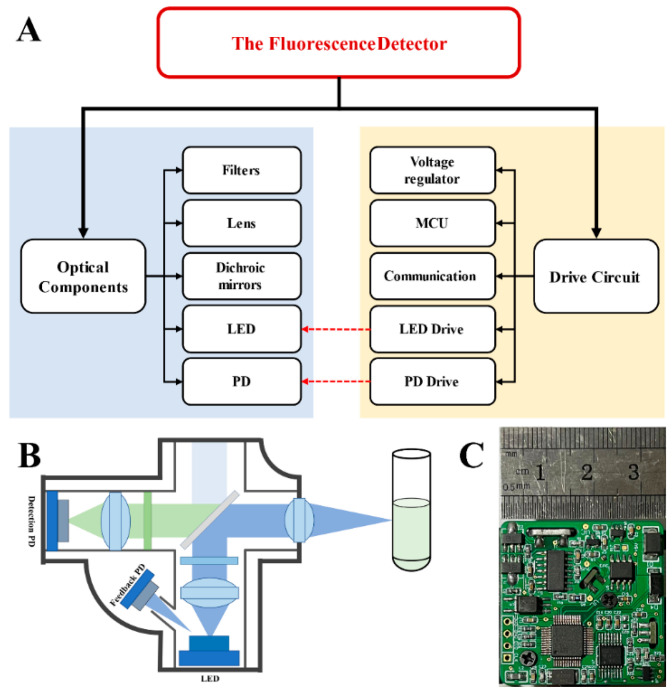
Overall structure of the detector: (**A**) a functional block diagram of the fluorescence detector, including optical components and drive circuit; (**B**) a schematic diagram of the optical path—blue indicates the excitation light, and green indicates the fluorescence emitted by sample; (**C**) a printed circuit board (PCB) picture of the fluorescence detector.

Figure 1A displays a functional block diagram of the detector. The whole detector consists of two parts: a confocal optical path and drive circuits. The confocal optical path includes optical elements and optical actuators. The optical elements contain lenses, filters, and dichroic mirrors for focusing, collimating, filtering, and separating excitation and emission light. The optical actuators comprise an LED, a PD, and a light intensity feedback PD. The mainstream excitation light sources are a xenon lamp, mercury lamp, laser, and high-power LED. Compared with other light sources, LED has the advantages of low price, small size, low energy consumption, and long life, which makes it more suitable as the excitation light source for portable devices [34,35]. Therefore, a high-power monochromatic LED (Golden DRAGON OSRAM GmbH, Munich, Germany) was chosen to be the excitation light source, the wavelength of which is 470 nm. In addition, considering cost and size, a more easily integrated high-sensitivity silicon PD (S1337-33BR, Hamamatsu Photonics Co., Ltd., Bridgewater, NJ, USA) was selected to collect fluorescence. The driving circuit part mainly includes a voltage regulation circuit, a microcontroller unit (MCU), and a communication unit, as well as an LED driving circuit and a photoelectric processing circuit, which are the focus of this paper.

Figure 1B shows the structure of the optical path; the LED excitation light was focused by a convex lens (GL12-006-006, Beijing Golden Way Scientific Co., Ltd., Beijing, China), filtered by a bandpass filter of 450~500 nm (BP-470, Shanghai Mega-9 Photoelectric Co., Ltd., Shanghai, China), then reflected by a long-pass dichroic mirror (DM-650, Shanghai Mega-9 Photoelectric Co., Ltd.) and, finally, focused onto the sample by a convex lens (GL11-006-008, Beijing Golden Way Scientific Co., Ltd.). The fluorescence emitted from the sample returns along the original path and can pass through the dichroic mirror due to the longer wavelength. The fluorescence was then filtered by a bandpass filter of 500~550 nm (BP-525, Shanghai Mega-9 Photoelectric Co., Ltd.), focused by the lens (GL12-006-006, Beijing Golden Way Scientific Co., Ltd.), and finally collected by the PD. In addition, another PD (BPW34BS, OSRAM GmbH) was used for light intensity feedback to help the circuit obtain a stable excitation light. Figure 1C shows a physical picture of the printed circuit board, which measures 35 mm × 35 mm.

**Figure 2:**
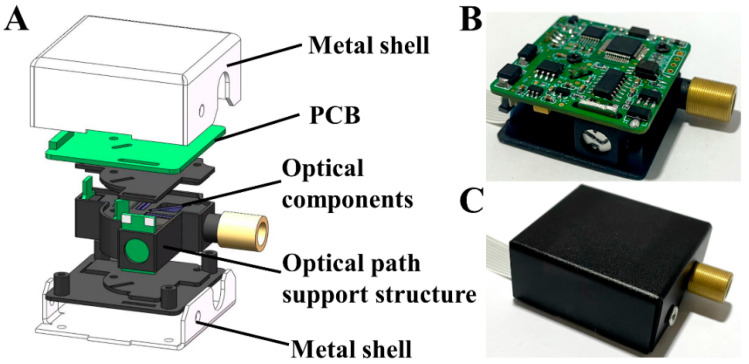
Structure and assembly of the fluorescence detector: (**A**) an exploded view of the three-dimensional (3D) structure; (**B**) a photo of the internal structure of the detector; (**C**) a photo of the detector with the metal box.

An exploded view of the three-dimensional (3D) structure and an internal physical photo of the detector are shown in Figure 2A,B, respectively. As can be observed in the figure, all the optical components are mounted in a 3D printed optical path support structure with a green printed circuit board (PCB) fitted to it as a compact whole, and this whole is then placed inside a metal box to shield it from electromagnetic noise. The size of the entire detector is only 40 mm × 40 mm × 18 mm; the actual photo is shown in Figure 2C.

### 2.2. Fluorescence Detector Circuit Design

#### 2.2.1. Detector Operating Principle

**Figure 3:**
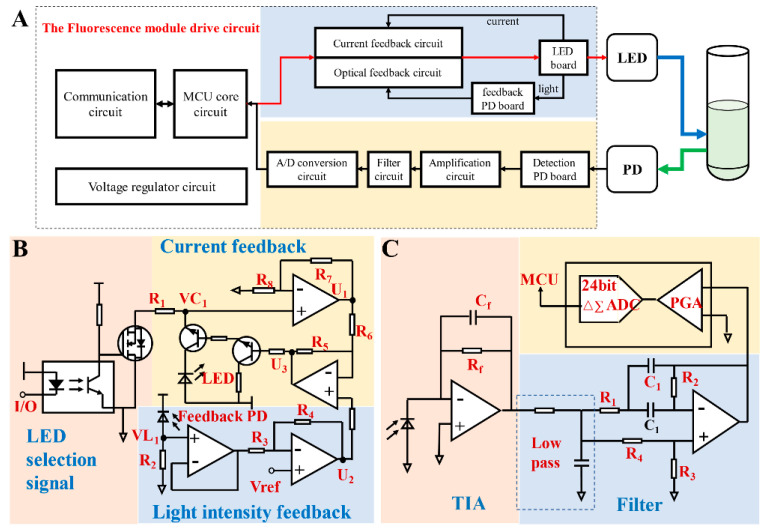
Overall circuit of the fluorescence detector: (**A**) a principle block diagram of the drive circuit; (**B**) the current–light dual negative feedback LED driving circuit schematic, including three parts: (1) current feedback, (2) light intensity feedback, and (3) LED selection signal; (**C**) photoelectric processing circuit schematic, including: transimpedance amplifier (TIA), filter, and analog-to-digital (A/D) conversion.

As mentioned before, the control circuit is the core of the detector, and Figure 3A shows a working principle diagram of the circuit. The MCU interacts with external devices for command and data through the communication unit to control the LED and send the fluorescence data. The light of the LED irradiates the sample through the confocal light path and emits fluorescence. The PD receives the returned fluorescence signal and converts it into an electrical signal. After amplifying, filtering, and analog–digital (A/D) converting, the data are sent to the MCU. A current–light dual negative feedback strategy was promoted to ensure the stability of excitation light. The drive current enters the current feedback circuit after the sample; the LED light is converted into a current by feedback PD and then enters the light intensity feedback circuit. These two negative feedbacks simultaneously regulate the LED to achieve stable unfluctuating excitation light. Because of the weak fluorescence signal and the lack of amplification ability of PD, the signal acquisition circuit uses a transimpedance amplifier circuit to amplify the signal and a filtering circuit to filter out the noise to improve anti-interference capability.

#### 2.2.2. The Current–Light Dual Negative Feedback LED Drive Circuit

As a semiconductor device, LED exhibits negative temperature characteristics that its forward voltage decreases with increasing temperature. Thus, temperature has a great influence on the performance of LED [36]. In addition, the volt-ampere characteristic of LED is nonlinear, and the working current and the applied voltage have an exponential relationship. When the forward voltage exceeds a certain threshold, the working current will increase sharply with an increase in the applied voltage. LED luminous intensity is determined by the drive current—too large a current will cause LED light decay, and too little a current will affect its luminous intensity, while only a constant current can ensure the reliability of the LED while achieving the desired luminous intensity [37]. Considering this, an active constant current compensation circuit is a definite requirement for a driving circuit. One of the advantages of LED is its long-life superiority over incandescent lamps. However, the luminous efficiency decreases with aging and changes with internal and external temperature [38]. Moreover, the brightness of LED is related to the drive current but also varies depending on the environment. Thus, light intensity feedback can be added to the current feedback to further obtain a stable excitation light source. There are some dedicated constant current drivers for LED on the market, but most of them regulate the output current through current sampling resistors, such as MAX16836, which is a buck constant current driver chip. To ensure the stability of the excitation light source, a current–light dual negative feedback LED drive circuit was designed to make the current and light intensity constant. Figure 3B shows a schematic of the circuit, which can be divided into three parts: (1) LED signal control—the signal from the MCU made by a MOSFET (YJL3400A, Yangzhou Yangjie Electronic Technology Co., Ltd., Yangzhou, China) was turned on or off by an optocoupler (PC410, Sharp Corp., Sakai, Japan). (2) Current feedback—the LED drive current was sampled by a resistor *R*_1_ and then connected to an operational amplifier (LMV324, Texas Instruments Inc.). Passing through two operational amplifiers, the current was adjusted by a triode. (3) Light intensity feedback—feedback PD generated a light current when receiving LED light, and this current was also sampled by a resistor *R*_2_. Similarly, it was regulated by the triode after passing the operational amplifier.

The voltage relationships at each node of the circuit are as follows:

(1) $$U_{1}=\left(1+\frac{R_{7}}{R_{8}}\right)\times VC_{1}$$

(2) $$U_{2}=\left(1+\frac{R_{4}}{R_{3}}\right)\times V_{ref}-\frac{R_{4}}{R_{3}}\times VL_{1}$$

(3) $$U_{3}=\left(1+\frac{R_{5}}{R_{6}}\right)\times U_{2}-\frac{R_{5}}{R_{6}}\times U_{1}$$

When the current flowing through the LED decreases, the voltage *VC*_1_ decreases and *U*_1_ decreases; from Equation (3), *U*_3_ increases, and the triode base current increases, so the LED current increases, thus achieving a constant current. On the other hand, when the light intensity of the LED decreases with the change in use time and temperature, the reverse current generated by the PD decreases, and the voltage *VL*_1_ decreases. Combining Equations (1) and (2), *U*_2_ increases, *U*_3_ increases, and LED current increases, thereby realizing the negative feedback of light intensity. In summary, the LED finally emits a stable excitation light under the dual regulation of two feedbacks: current and light intensity.

#### 2.2.3. The Photoelectric Processing Circuit

The fluorescence emitted by an excitation light is generally very weak, and the order of magnitude is around nW. Therefore, it is necessary to choose a highly sensitive silicon PD for the sensitive collection of fluorescence. Table 1 presents some available PDs on the market and their optical characteristics. Considering size and sensitivity, we chose S1337-33BR (Hamamatsu Pho-tonics Co., Ltd., Hamamatsu, Japan) with a sensitivity of 500 nm~700 nm, which is bigger than 0.3 A/W. Since PD has no ability to amplify the signal, and an electrical signal is also accompanied by noise, the fluorescent signal acquisition circuit needs to be amplified and filtered to obtain a high-amplitude electrical signal and low noise [39,40]. The operational amplifier is the key point of the photoelectric processing circuit, and an operational amplifier with a low noise level should be selected because the detection limit of PD with an amplifier is determined by its noise characteristics. Therefore, we chose a low-noise operational amplifier (LTC6240, Analog Devices Inc., Wilmington, MA, USA) that is practical for PD amplification circuits.

Figure 3C is a schematic of the photoelectric signal processing circuit, which mainly includes three parts: photoelectric conversion, amplification, filtering, and analog-to-digital conversion. Compared with applying reverse bias in photoconductive mode, PD has worked in photovoltaic mode and can reduce the dark current [41,42]. In this circuit, the light current generated by PD was converted to voltage through a transimpedance amplifier (TIA), and the current–voltage ratio was determined by a resistor *R_f_* [43]. A resistor-capacitor (RC) low-pass filter and notch filter were used to remove high-frequency noise and 50 Hz industrial frequency interference. Finally, the analog signal, after filtering, was converted to a digital signal by an A/D converter (ADS1220, Texas Instruments Inc., Dallas, TX, USA) and was sent to the MCU for subsequent operations.

The ideal photodiode can be considered a constant current source with good output characteristics when its load resistance is zero. According to the characteristics of the ideal operational amplifier, the input impedance of the preamplifier circuit in Figure 3 is:

(4) $$R_{in}=R_{f}/\left(1+A\right)$$

In Formula (4), *A* is the open-loop gain of the operational amplifier, and *R_f_* is the feedback resistance. Usually, *A* ≥ 10^6^, so *R_in_* ≈ 0, which guarantees the linear operating characteristics of the PD in photovoltaic mode. The weak photocurrent forms a voltage drop through a large feedback resistor *R_f_*, thus realizing the *I–V* conversion of photocurrent to voltage. The greater the feedback resistance, the greater the output voltage, but there is also an upper limit to the choice of the resistance—too large a feedback resistance causes the circuit to generate self-excited oscillations. Through the parallel capacitor *C_f_*, it can play the role of phase compensation and prevent self-excitation, increasing the stability of the circuit.

In order to filter the high-frequency noise in the signal and the industrial frequency interference in the mains, the transimpedance amplified signal was filtered by a simple first-order RC low-pass filter circuit and a notch circuit for 50 Hz. This notch filter was a bandpass bridge circuit consisting of a single operational amplifier (LMV321, Texas Instruments Inc.) with four resistors and two capacitors. The transfer function of this trap circuit is:

(5) $$A_{us}=A_{up}\times \frac{s^{2}+s\left(\frac{2}{R_{2}C}-\frac{1}{R_{3}C}\right)+\frac{1}{R_{1}R_{2}C^{2}}}{s^{2}+2s\frac{1}{R_{2}C}+\frac{1}{R_{1}R_{2}C^{2}}}$$

where *s* is the complex frequency, *A_up_* is the passband amplification, and $\omega_{n}=1/R_{1}R_{2}C^{2}$ is the center frequency, which can be changed by selecting *R*_1_, *R*_2_, and *C*_1_. Herein, the center frequency is 50 Hz because of the filtering of the industrial frequency interference.

### 2.3. A Test Platform Based on the Fluorescence Detector

**Figure 4:**
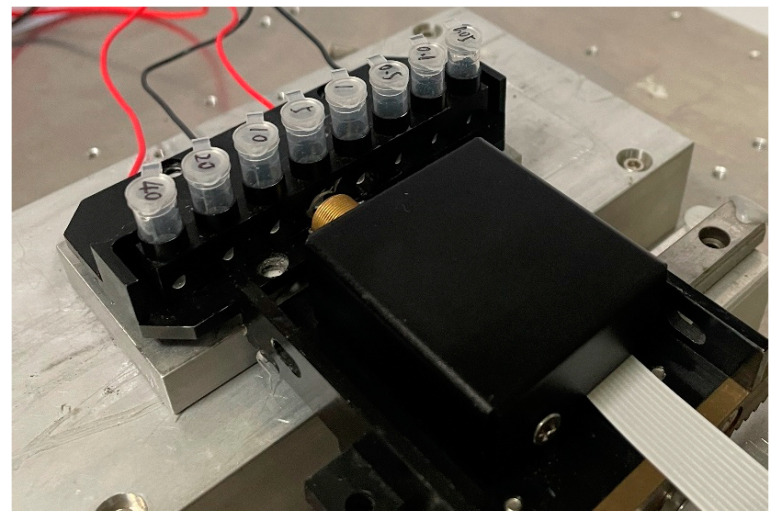
Photo of the test platform based on the fluorescence detector in this paper.

Based on the fluorescence detector in this paper, a test platform (Figure 4) was built to perform a series of performance tests to evaluate the detector. As shown in Figure 4, the test platform contains an eight-channel metal heat trap with two TECs installed under it to realize PCR thermal cycling control. A step motor can drive the fluorescence detector to scan the eight channels sequentially and obtain the corresponding fluorescence signals. On this platform, a *fluorescein sodium* concentration gradient test and a real-time fluorescence PCR test for *Legionella pneumophila* were performed.

The *fluorescein sodium* concentration gradient test was performed to evaluate the linearity and sensitivity of the detector. The reagent used was analytical grade *fluorescence sodium* (Shanghai Maikun Chemical Co., Ltd. Shanghai, China) and was configured to a solution with 1 mM concentration using ultrapure water. Then, we diluted the 1 mM solution to the following concentrations: 0.05 nM, 0.1 nM, 0.5 nM, 1 nM, 5 nM, 10 nM, 20 nM, 40 nM, 80 nM, 100 nM, 150 nM, 200 nM, 300 nM, and 400 nM. A total of 80 μL of each solution was loaded into a high-permeability PCR tube (PCR-02-L-C, ASYGEN). Then, those tubes were placed on the built test platform for fluorescence detection by the detector in sequence. As a control, a spectrofluorophotometer (RF-6000, SHIMADZU) was used for the same test, setting the excitation light at 470 nm and the emission light range from 485 nm to 600 nm. In addition, a cuvette (10 mm × 10 mm) was used as the sample cell, and the solution volume was 200 μL.

The real-time PCR test on the built test platform was performed to verify the practical application capability of the fluorescence detector. To make the results more convincing, a commercial PCR system (StepOnePlus, Applied Biosystems, Waltham, MA, USA) was used as a standard control. The *Legionella pneumophila* (LP) Nucleic Acid Test Kit (Z-RD-0057-02, Shanghai Zhijiang Biotechnology Co., Ltd., Shanghai, China) and the positive control sample in this kit were used for this experiment. The corresponding fluorescence channel was FAM. The real-time PCR contents are shown in Table 2. The reaction conditions were predenaturation at 94 °C for 120 s, as well as 40 cycles of denaturation at 93 °C for 15 s and annealing extension at 60 °C for 60 s. The fluorescence signal was measured at the end of each cycle.

## 3. Results and Discussion

### 3.1. LED Light Intensity and Stability Testing

**Figure 5:**
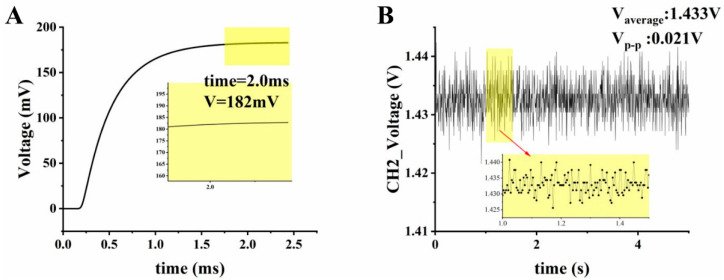
LED stability test result: (**A**) Multisim simulation of voltage value across the LED current sampling resistor; (**B**) oscilloscope waveform of voltage value across the LED current sampling resistor.

To verify the feasibility of the LED driving circuit, simulations were performed by NI Multisim 14.0. The feedback PD was equivalent to a 1 μA current source and constituted a feedback voltage *VL*_1_ with the resistor *R*_2_. The output used the current clamp tool in the software to convert the LED current to voltage, and the ratio of current to voltage was 1 mV/mA. Figure 5A shows the simulation of its oscilloscope, and it can be observed that the voltage tended to stabilize after 2.0 ms, and the LED current was stable at 182 mA. In addition, the voltage fluctuations across the current sampling resistor in the actual circuit were measured using an oscilloscope (VirtualBench, National Instruments Corp., Austin, TX, USA) after lighting the LED. Figure 5B shows the voltage waveform on the current sampling resistor, with an average voltage value of 1.433 V, a peak-to-peak value of 0.021 V, and a voltage fluctuation of ±0.7%.

**Figure 6:**
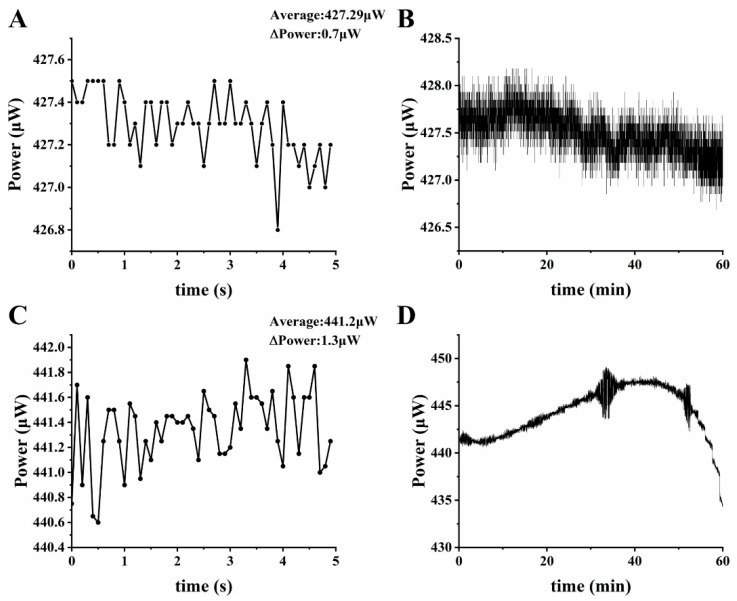
Comparison of the luminous power of LEDs driven by two different LED driving circuits: the LED fluctuation in 5 s (**A**) and 1 h (**B**) driven by the dual negative feedback circuit built in this paper, and the LED fluctuation in 5 s (**C**) and 1 h (**D**) driven by a LED driver (MAX16836).

A LED constant current driving circuit was built based on MAX16836, and the luminous power of the LEDs driven by the MAX16836-based circuit and the dual negative feedback circuit in this paper were measured by an optical power meter (PM320E, Thorlabs Inc., Newton, NJ, USA), respectively. For each circuit, the LED luminous power in short time (5 s) and long time (1 h) were tested. Figure 6A,C shows the LED fluctuations of the dual negative feedback circuit, while Figure 6B,D shows the LED fluctuations of the MAX16836-based circuit. As we can see, the dual negative feedback circuit in this paper showed superior stability whether in short time or in long time. The fluctuation within 5 s was 0.7 μW ± 0.08%, and the fluctuation within 1 h was less than 2 μW.

Fluorescence is the light emitted by a substance after absorbing light or other electron radiation [44,45], and the relationship between fluorescence intensity *F* and excitation intensity *I*_0_ is:

(6) $$F=\varphi I_{0}\left(1-10^{-\varepsilon lc}\right)$$

In Formula (6), *φ* is the fluorescence quantum yield, *ε* is the molar absorption coefficient, *l* is the thickness of the liquid pool, and *c* is the concentration of the fluorescent substance. It can be seen from the above formula that the fluorescence intensity is proportional to the excitation light intensity when the concentration is constant, so the fluctuation of the excitation light source will directly affect the stability of the detected fluorescence signal, thereby affecting the accuracy of the detection result. In other words, due to the double regulation of the LED by the current–light intensity dual negative feedback circuit, the LED light source of the detector can meet the requirements of stable light intensity and small fluctuations.

### 3.2. Fluorescein Sodium Solution Concentration Detection Experiment

**Figure 7:**
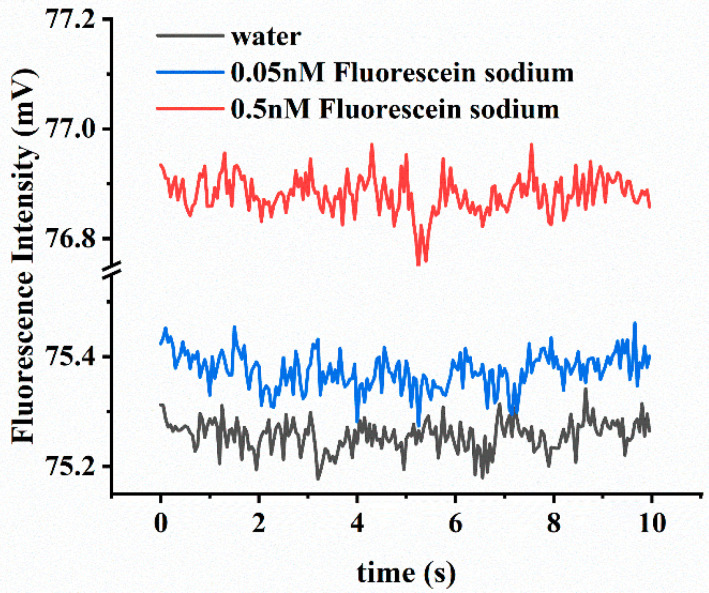
The fluorescence curves of water and sodium fluorescein solutions with concentrations of 0.05 nM and 0.5 nM obtained by the detector.

To better demonstrate the stability of the detector, for each *fluorescein sodium* solution, we detected it for 10 s. Figure 7 shows the complete fluorescence curves in 10 s of ultrapure water and *sodium fluorescein* solutions with concentrations of 0.05 nM and 0.5 nM. As can be observed, the fluorescence signal collected by the fluorescence detector fluctuated very little, all within 0.5 mV (<±0.35%). Moreover, the signal value of 0.05 nM concentration was still higher than that of the blank control (ultrapure water), indicating that the detector had a very sensitive response.

**Figure 8:**
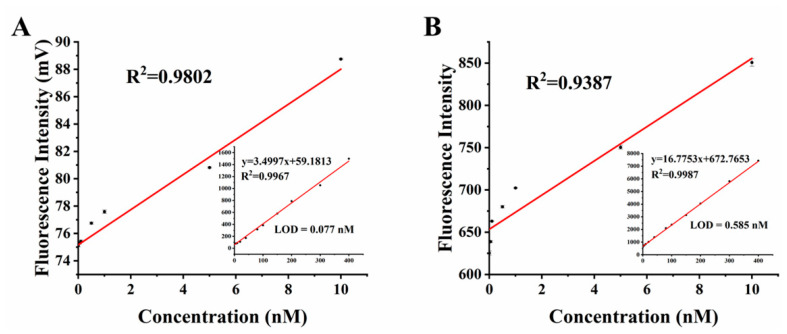
Linearity of the fluorescence signal with the concentration of *fluorescein sodium* solution: (**A**) the results from the detector in this paper; (**B**) the results from the spectrofluorophotometer.

For further analysis, we processed the raw data. For the 10 s fluorescence values of each concentration solution measured by this detector, we averaged them as the final value. As for the spectrofluorophotometer, we averaged the peak values of spectral data (512 nm~514 nm) as the final value. Figure 8 demonstrates the linearity between the concentration of *fluorescein sodium* and the fluorescence signal. Compared with the spectrofluorophotometer, our detector also showed good linearity at low concentrations (R^2^ = 0.9802). Since the concentration and signal value may not be linear in the vicinity of a blank sample, the LOD of the sample is considered the concentration at which the detection signal is equal to three times the standard deviation of the blank sample measurement. The formula for LOD is as follows:

(7) LOD=3δ/k

where *δ* is the standard deviation of the blank sample (10 repeated measurements), and *k* is the slope of the linear response curve. The LOD of our fluorescence detector for *fluorescein sodium* was 0.077 nM, while the LOD of the spectrofluorophotometer was 0.585 nM, which also indicated that it had a fairly high sensitivity.

### 3.3. Real-Time Fluorescence PCR Test for Legionella pneumophila

**Figure 9:**
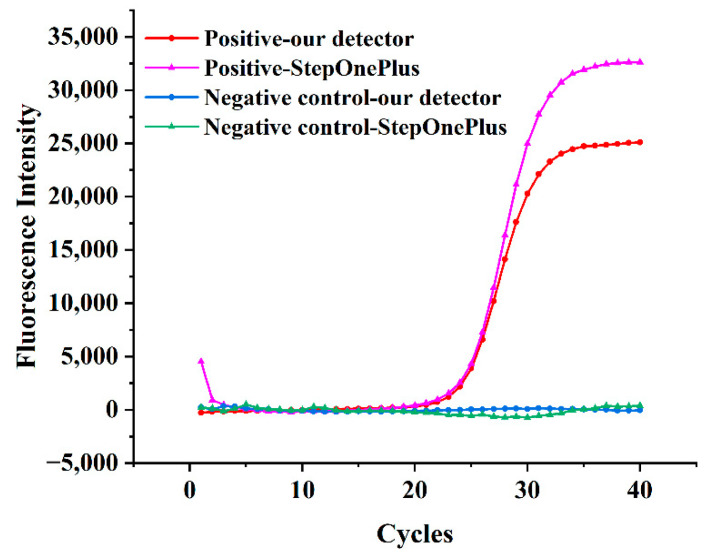
Real-time fluorescence PCR curves for detection of *Legionella pneumophila* and negative control from our detector and StepOnePlus system.

Figure 9 shows the real-time PCR curves for the detection of *Legionella pneumophila* and the negative control from our detector and StepOnePlus system, in which the x-axis is the number of cycles and the y-axis is the fluorescence intensity. As we know, the results of real-time PCR come from the analysis and calculation of the fluorescence curves. Any shift and fluctuation in the curves can affect the accuracy of the assay results. In order to obtain accurate real-time PCR curves, the fluorescence detector must have stable excitation light and accurate emission light acquisition. In Figure 9, the curve from our detector was smooth without any fluctuation, and the curves of both platforms were similar in shape. Thus, the feasibility of this detector for real-time fluorescence PCR applications was initially validated.

## 4. Conclusions

The accurate measurement of biological and chemical substances has a very important role in applications such as environmental monitoring, clinical diagnosis, and pathogen detection. Fluorescence detection can quantitatively and qualitatively analyze targets by exciting samples to emit fluorescence. Due to the advantages of high sensitivity and accuracy, there is an increasing demand for portable fluorescence detection instruments in various applications. In this paper, we have developed a highly sensitive and highly integrated fluorescence detector. This detector is based on a confocal optical path structure with LED as the light source and PD as the photodetector. The drive circuit and optical components are all integrated into a small metal box (40 mm × 40 mm × 18 mm). We concentrated our efforts on LED drive and photoelectric processing circuits, which are the key and difficulty of fluorescence detection. We analyzed the topology of the LED drive circuit, and proposed the idea of the dual negative feedback control of current and light intensity and carried out the design and simulation of the circuit. As for photoelectric processing, we designed a circuit, including a PD amplifier, filter, and A/D converter, to obtain weak fluorescence signals precisely.

We tested the working current of LED and measured the luminous power of LED during short-time (5 s) and long-time (1 h) working, respectively. Compared with the MAX16836-based constant current driving circuit, the dual negative feedback circuit in this paper showed superior stability whether in short time or in long time. In addition, a test platform based on the fluorescence detector was built to perform a *fluorescein sodium* concentration gradient test and a real-time fluorescence PCR test for *Legionella pneumophila*. Benefiting from the high gain and low noise of the signal acquisition circuit, the detector had high sensitivity and linearity. The real-time PCR fluorescence curve from our detector was smooth without any fluctuation, and it was similar to the curves of the commercial PCR system in shape, which demonstrated its feasibility for real-time PCR.

Currently, the light intensity of this fluorescence detector cannot be adjusted by the user. To make the detector suitable for more applications that require different excitation light intensities, we will add this function in our next work. On the other hand, the current single-channel detector cannot meet the demand for multiple testing; in the future, we will develop a multichannel detector based on this work.

## Figures and Tables

**Table 1:** Alternative PDs and optical characteristics.

Manufacturer	PD	Spectral Response Range (nm)	Peak Sensitivity Wavelength (nm) λ	Photosensitivity (A/W) λ
Hamamatsu Photonics	S1337-33BR	340 to 1100	960	0.62
	S1133-14	320 to 1000	720	0.40
	S1227-33BR	190 to 1000	720	0.43
	S2387-33R	340 to 1100	960	0.58
OSI Optoelectronics	PIN-13DSB	350 to 1100	960	0.52
First Sensor	PS7-5B-TO5	350 to 1000	750	0.42
	PS11.9-5-TO5	400 to 1100	800	0.52

**Table 2:** Real-time PCR contents.

Reagents	Volume
LP Nucleic Acid Fluorescent PCR Assay Mix	36 μL
Enzyme (Taq + UNG)	0.4 μL
LP Positive Control	2 μL

## Data Availability

Data are contained within the article.
